# A retrospective study of the anterolateral thigh perforator flap in the treatment of chronic osteomyelitis of the leg with skin defects

**DOI:** 10.1016/j.jpra.2024.07.006

**Published:** 2024-07-22

**Authors:** Zhegang Zhou, Longbiao Yu, Fanbin Meng, Jingjing Wen, Yingfeng Xiao, Shengxiang Wan, Jing Yan, Hui Zeng, Fei Yu

**Affiliations:** a Department of Hand & Microsurgery, Peking University Shenzhen Hospital, China; b Department of outpatient operating room, Peking University Shenzhen Hospital, China; c Department of Orthopedics, Shenzhen Second People's Hospital, China; d Department of Bone & Joint Surgery, Peking University Shenzhen Hospital, China; e National & Local Joint Engineering Research Center of Orthopaedic Biomaterials, China; f Shenzhen Key Laboratory of Orthopaedic Diseases and Biomaterials Research, China

**Keywords:** Chronic osteomyelitis, Skin defect, Anterolateral thigh perforator flap, Retrospective study

## Abstract

**Background:**

As a chronic inflammatory process, chronic osteomyelitis is caused by bacterial infections that lead to bone destruction. This disease is more common in patients with open fractures and those undergoing multiple surgical procedures after trauma. We aimed to provide a comprehensive overview and critical assessment of the therapeutic efficacy of the anterolateral thigh (ALT) perforator flap in the management of chronic osteomyelitis with dermatologic and soft tissue imperfections localized in the lower extremity.

**Methods:**

A retrospective analysis involving a cohort of 16 patients who underwent ALT perforator flap reconstruction for the management of chronic osteomyelitis in the calf region that manifested with integumentary deficiencies was conducted.

**Results:**

During the follow-up period spanning from 4 months to 2 years, all 16 patients who underwent ALT perforator flap transplantation exhibited flap viability. Among these cases, 15 patients made a full recovery from the infection and 1 patient had partial survival. Among the 15 cases, 2 patients developed vascular crisis (owing to venous thrombosis during surgical exploration). One patient had a relapse of the disease 1-year post-surgery. The success rate of this surgical method was 15/16, and the surgical complications included flap crisis, flap necrosis, delayed wound healing, and recurrence of infection.

**Conclusion:**

The ALT perforator flap, which can cover bone and soft tissues and effectively control infections, can be applied to the treatment of chronic osteomyelitis of the lower limbs with skin defects. Overall, the muscle flap fills the dead space and medullary cavity and skin flap covers the skin defect.

## Introduction

Chronic osteomyelitis, a persistent inflammatory condition, is characterized by bone destruction resulting from bacterial infections.^1^ This pathology is frequently encountered in patients with open fractures^2^ or those who underwent multiple surgical procedures after trauma.^3^ Chronic osteomyelitis can be divided into hematogenous,^4^ traumatic,^5^ and exogenous^6^ subtypes based on its underlying etiology. Typically, chronic osteomyelitis management involves a combination of surgical interventions^7^ along with systemic or local antibiotics therapy.^8^ This debilitating condition predominantly affects young and middle-aged males across various age groups, with higher incidences observed in the tibia and femur regions. Notably, the lower leg is the most commonly afflicted area.^9^ Within the field of orthopedics, chronic osteomyelitis of the lower limbs presents as a frequent and challenging clinical entity.^10^ Patients suffering from this condition often develop recalcitrant wounds due to recurrent infections that manifest as sinuses, skin defects, exposed bones and internal implants, and bone scars. Chronic osteomyelitis often has a prolonged disease course, accompanied by elevated rates of treatment failure and recurrence.^11^ The anterolateral thigh (ALT) flap can be considered as an improved perforator flap prepared based on the perforator blood vessels that originate from the descending branch of the lateral femoral artery and pass through the lateral femoral muscle to reach the skin.^12^ The subcutaneous adipose tissue within this flap can be sculpted to fill tissue defects, with thinning of the fatty layer enabling the formation of a subdermal vascular network. This versatile flap can be microsurgically anastomosed to adjacent tissues or employed for vascular bridging that is commonly used in orthopedic reconstruction and repair procedures.^13^ In early clinical practice, the integration of the ALT perforator flap with staged debridement has emerged as a promising strategy yielding favorable outcomes in the management of chronic osteomyelitis. Therefore, this retrospective investigation aimed to assess the therapeutic efficacy of ALT perforator flaps in the treatment of chronic osteomyelitis of the leg with skin and soft tissue defects.

## Materials and methods

### General information

In this study, a total of 16 patients (including 11 men and 5 women, aged 16–62 years, with an average age of 37 years) who received treatment in our hospital from September 2008 to November 2020 were included (Table 1). The etiological factors for the injuries were diverse, with 9 patients sustaining injuries from traffic accidents, 3 patients from machine strangulation, 3 patients from heavy objects, and 1 patient from a fall. The duration of the diseases ranged from 3 months to 10 years and number of surgical procedures following the injuries varied from 2 to 9 times. Specifically, 6 patients presented with open wounds, including 1 patient with allograft bone infections exposed at the bone defect, and 10 patients suffered from chronic sinus drainage.

**Table 1:** Demographic data of all cases.

Case number	Age, years	Gender	Marital status	Educational level	Occupation	Diagnosis	Comorbidities	Hospitalization, days	Number of surgeries	Expense,
1	30	Female	Married	University	Professional technical personnel	Left ankle joint open comminuted fracture	None	25	3	35,762
2	35	Male	Married	Senior high school	Professional technical personnel	Post-operative right tibiofibular comminuted fracture with right tibia osteomyelitis	None	36	5	53,874
3	34	Female	Married	Senior high school	Professional technical personnel	Right tibiofibular osteomyelitis, residual necrosis after amputation	None	27	1	27,943
4	36	Male	Married	Junior high school	Farmer	Left tibia and fibula open comminuted fracture (Gustilo IIIB)	Infection	26	4	33,967
5	62	Male	Married	Senior high school	Insurance officer	Left tibia and fibula open comminuted fracture (Gustilo IIIC)	Infection	57	5	97,246
6	40	Female	Married	Primary school	Unemployed	Right calf osteomyelitis with soft tissue defect	None	22	2	36,479
7	40	Male	Married	Illiteracy	Unemployed	Left calf chronic osteomyelitis	None	26	1	32,387
8	34	Male	Married	Junior high school	Professional technical personnel	Left medial ankle soft tissue defect, left tibial osteomyelitis	None	25	2	47,382
9	42	Male	Married	Junior high school	Professional technical personnel	Left ankle infection	None	37	3	58,494
10	40	Male	Married	Junior high school	Loading and unloading worker	Right calf incomplete detachment injury	Infection, soft tissue necrosis	36	2	49,723
11	38	Male	Married	Primary school	Farmer	Right fibular osteomyelitis	None	23	2	38,464
12	40	Male	Married	Junior high school	Professional technical personnel	Left ankle osteomyelitis, soft tissue defect	None	37	2	28,934
13	42	Female	Married	Senior high school	Professional technical personnel	Right ankle osteomyelitis, soft tissue defect	None	27	3	55,623
14	16	Female	Unmarried	Senior high school	Student	Post-operative infection and necrosis of left tibiofibular fracture	None	16	2	38,384
15	33	Male	Married	Senior high school	Professional technical personnel	Left knee joint open fracture with dislocation, soft tissue necrosis and infection after popliteal artery and vein transplantation repair surgery	Infection, soft tissue necrosis	104	11	23,5768
16	30	Male	Married	University	Professional technical personnel	Post-operative infection and soft tissue defect of right tibiofibular fracture	None	19	2	49,638

The patients were classified using the Cirny–Mader classification according to the *Chinese Expert Consensus on the Diagnosis and Treatment of Infection after Internal Fracture Fixation (2018).* Based on the anatomical classification criteria, 12 patients were categorized as type IV grade B and 4 patients as type IV grade C. Based on the host classification criteria, 14 patients were classified as type B and 2 patients as type C. Bacterial culture results showed that 6 patients had gram-negative infections, 2 had gram-positive infections, and 8 had mixed infections. Preoperative color Doppler ultrasound scan or digital subtraction angiography (DSA) examinations identified that 7 patients suffered from anterior tibial vascular injuries and 1 patient had posterior tibial vascular injuries. The area of skin and soft tissue defects after expansion ranged from 4 cm × 6 cm to 14 cm × 23 cm, whereas the area of the grafted flap ranged from 14 cm × 7 cm to 25 cm × 16 cm.

### Surgical methods

All patients underwent staged surgical interventions.

#### First-stage lesion expansion

In the initial stage, debridement was undertaken to address the sinus or wound via the removal of necrotic and infected tissues and dead bones through a widened incision. Subsequently, the samples were collected from multiple sites within the lesion for bacterial culture and drug susceptibility testing. In cases with internal fixation, it was either removed or replaced with an external fixation frame. Following this, a reamer was employed to enlarge the medullary cavity or a curette was used to remove the inflammatory biofilm from the medullary cavity. Subsequently, a window of <1/3 of the diameter and width of the backbone was opened. The wound was thoroughly cleaned with normal saline, hydrogen peroxide, and diluted compound iodine, and cleaning was repeated until the normal bones and soft tissues were reached. Then, a vacuum drainage (VSD) sleeve silicone drainage tube was inserted into the deep layer of the soft tissue or medullary cavity to serve as the lavage inlet tube. Multiple pieces of VSD are placed in layers to eliminate the dead space in deep wounds. After the patient is transferred to the ward, antibiotic saline is administered for continuous and slow perfusion and flushing.

#### Second-stage bone and skin defect repair

The treatment of patients with varying sensitivity to vancomycin was based on the results of bacterial culture and drug susceptibility experiments. In cases where patients exhibited sensitivity to vancomycin, it was homogeneously combined with bone fillers at a concentration of 20% to fabricate granules, which were subsequently inserted into the medullary cavity. Conversely, for patients who were insensitive to vancomycin, proper antibiotics were selected and mixed with absorbable artificial bone materials at a concentration of 20%. Following coagulation, these granules were prepared and packed into the medullary cavity. Patients who tested negative for bacteria were treated with vancomycin artificial bone granules. An alternative approach involved re-surgery to obtain materials for cultivation. The selection of the aforementioned methods was contingent upon the relevant results. In instances where fractures or bony instabilities occurred post-expansion, stabilization was achieved using an external fixator. Ultimately, the wound was reconstructed and covered by transferring the ALT perforator free flap.

#### Surgical techniques

Thorough debridement was necessary. Moreover, hardened and dead bones needed to be thoroughly removed until a red pepper sign appeared. After debridement, drainage and NPWT were performed and were repeated until the bacterial cultures were negative. The flap was cut 20–30% larger than the affected area and the pedicle was taken away as far as possible from the infected area. A ratio of 1 artery and 2 veins chosen with the application of drain, vancomycin, calcium sulfate, and sufficient antibiotics.

## Results

**Figure 1:**
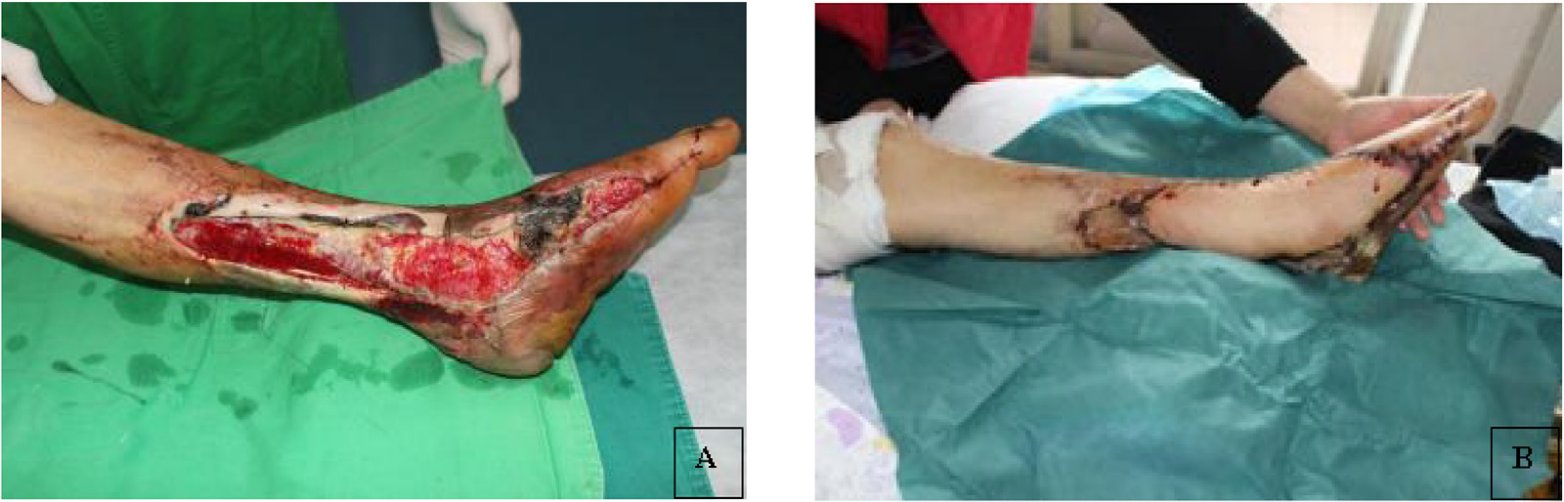
Photos of a patient's lower limb before and after surgery.

**Figure 2:**
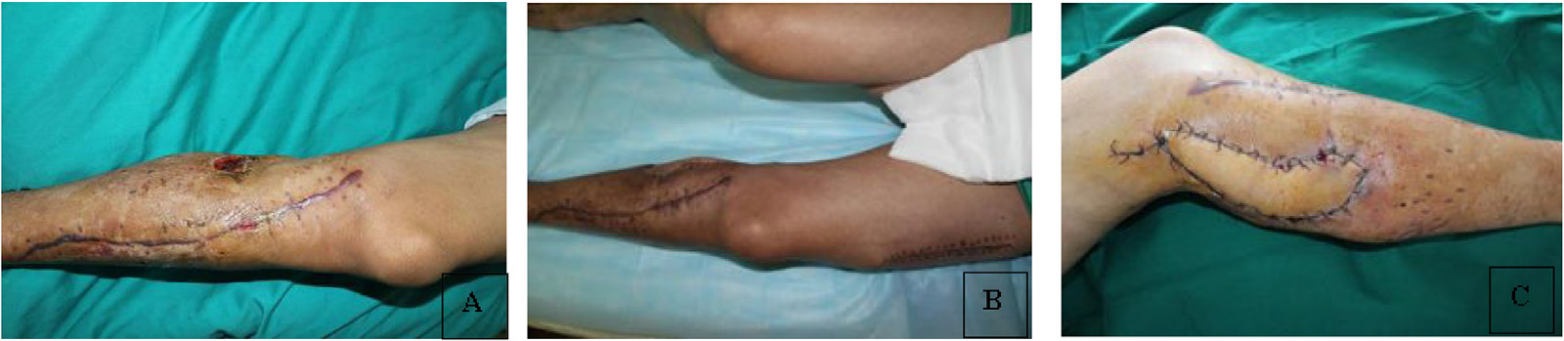
Partial necrosis of the skin flap.

The study comprised 16 patients (Figure 1) with outcomes of complete flap survival in 15 cases and partial survival in 1 case (Figure 2). Among the 15 patients with complete flap survival, 2 experienced venous crisis within 48 h post-operatively. These 2 patients had experienced venous thrombosis during surgical exploration. Flap of 1 patient survived fully after venous anastomosis. In another patient, the surrounding tissue was compressed owing to edema, and the edema was relieved after partial suture removal, dehydration, and swelling treatment. However, the wound could not be sutured and 1/3 of the distal skin flap was necrotic, which was repaired by skin grafting 2 weeks after surgery. Post-operative follow-up duration ranged from 4 to 24 months. The grafted artificial bone and the tibia and fibula exhibited complete healing within 6 to 12 months post-surgery. Notably, recanalization of the tibial marrow cavity was observed in 1 patient 16 months post-operatively. Additionally, 1 patient experienced disease recurrence 1 year post-surgery, which was successfully managed through debridement, infection control, and wound healing.

## A typical patient

**Figure 3:**
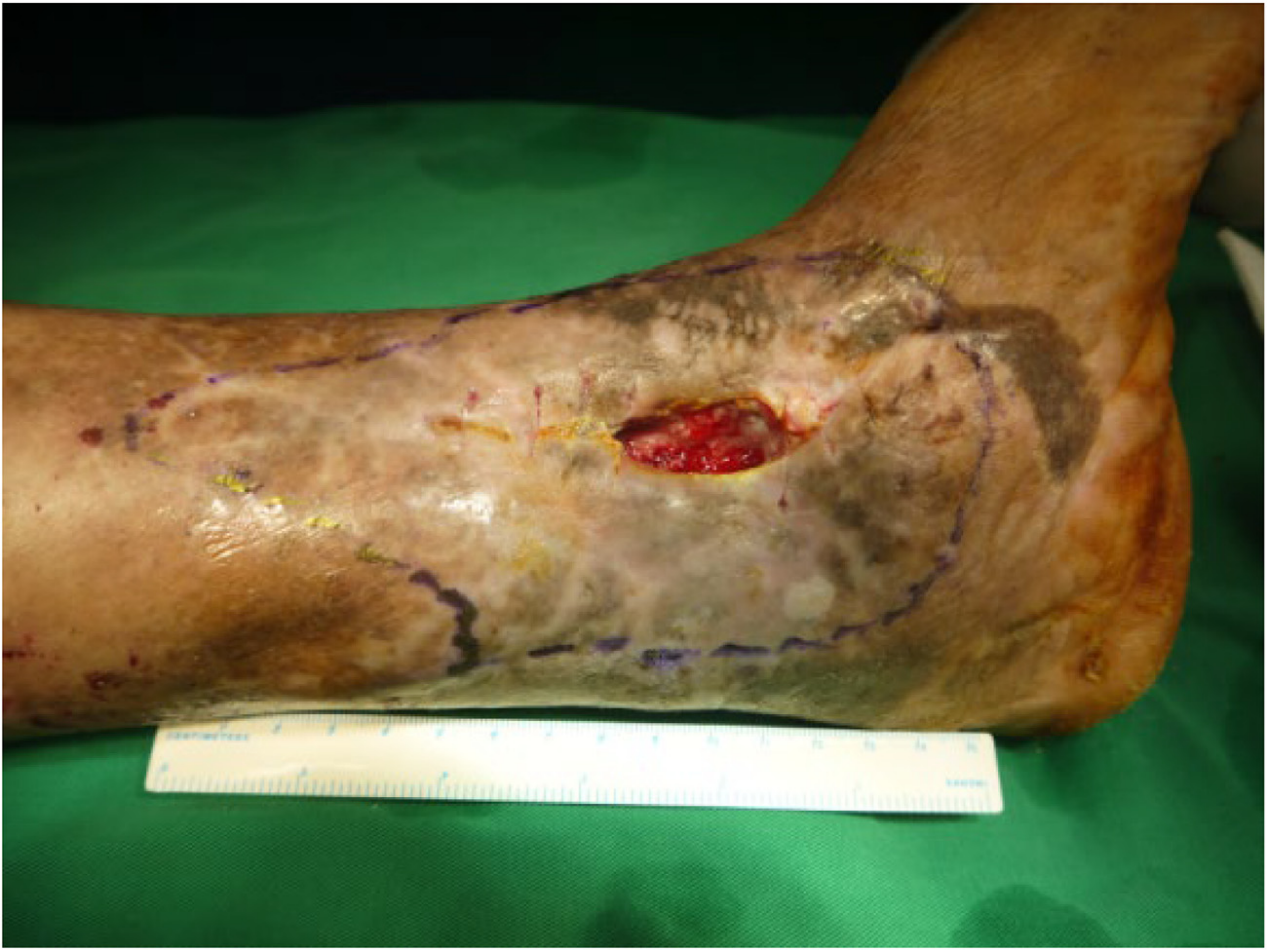
Preoperative image of a 5 cm × 6 cm skin defect at the medial malleolus of the right calf with exposed tibia.

**Figure 4:**
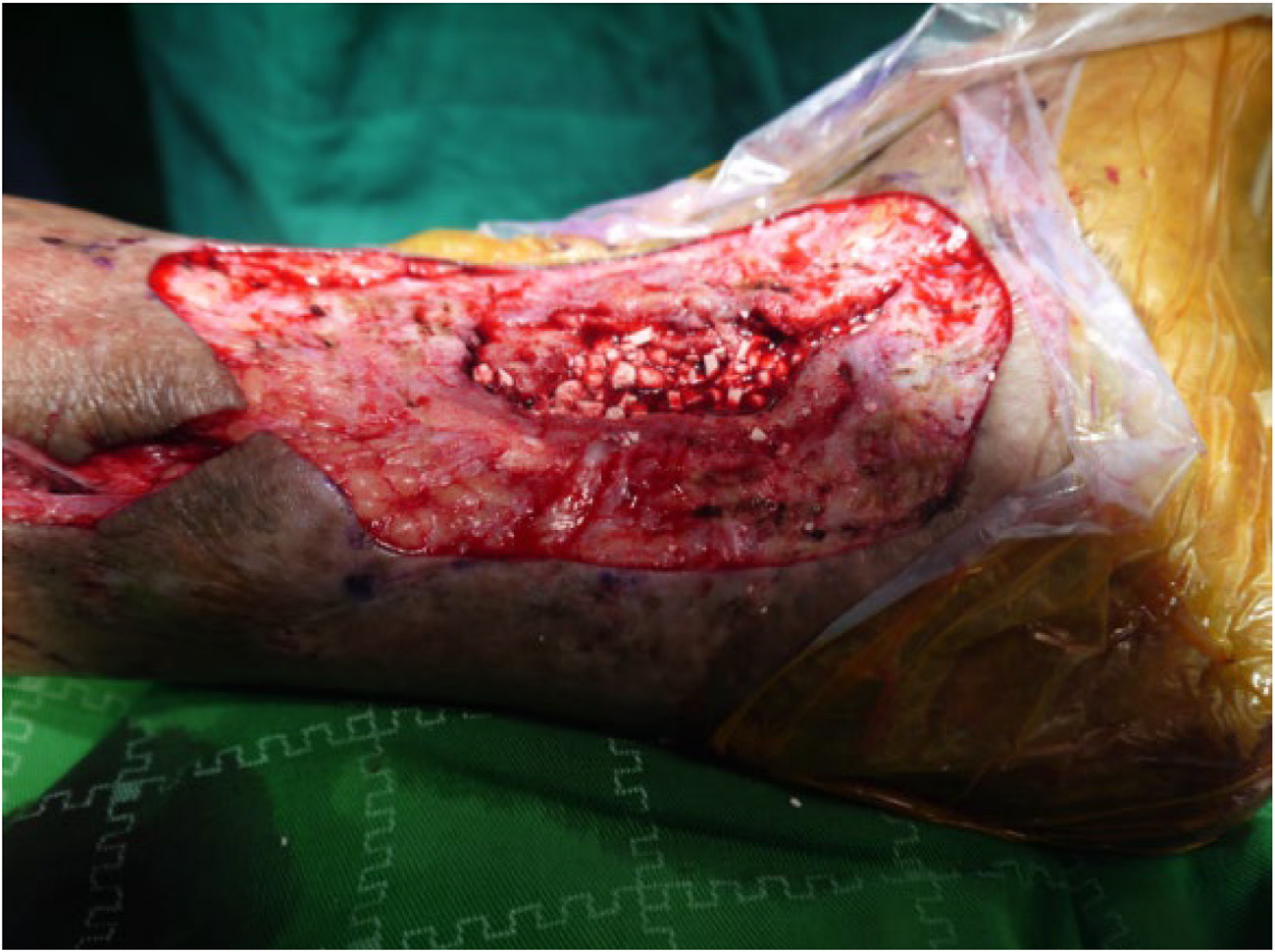
Artificial bone mixed with vancomycin is implanted into the bone marrow cavity.

**Figure 5:**
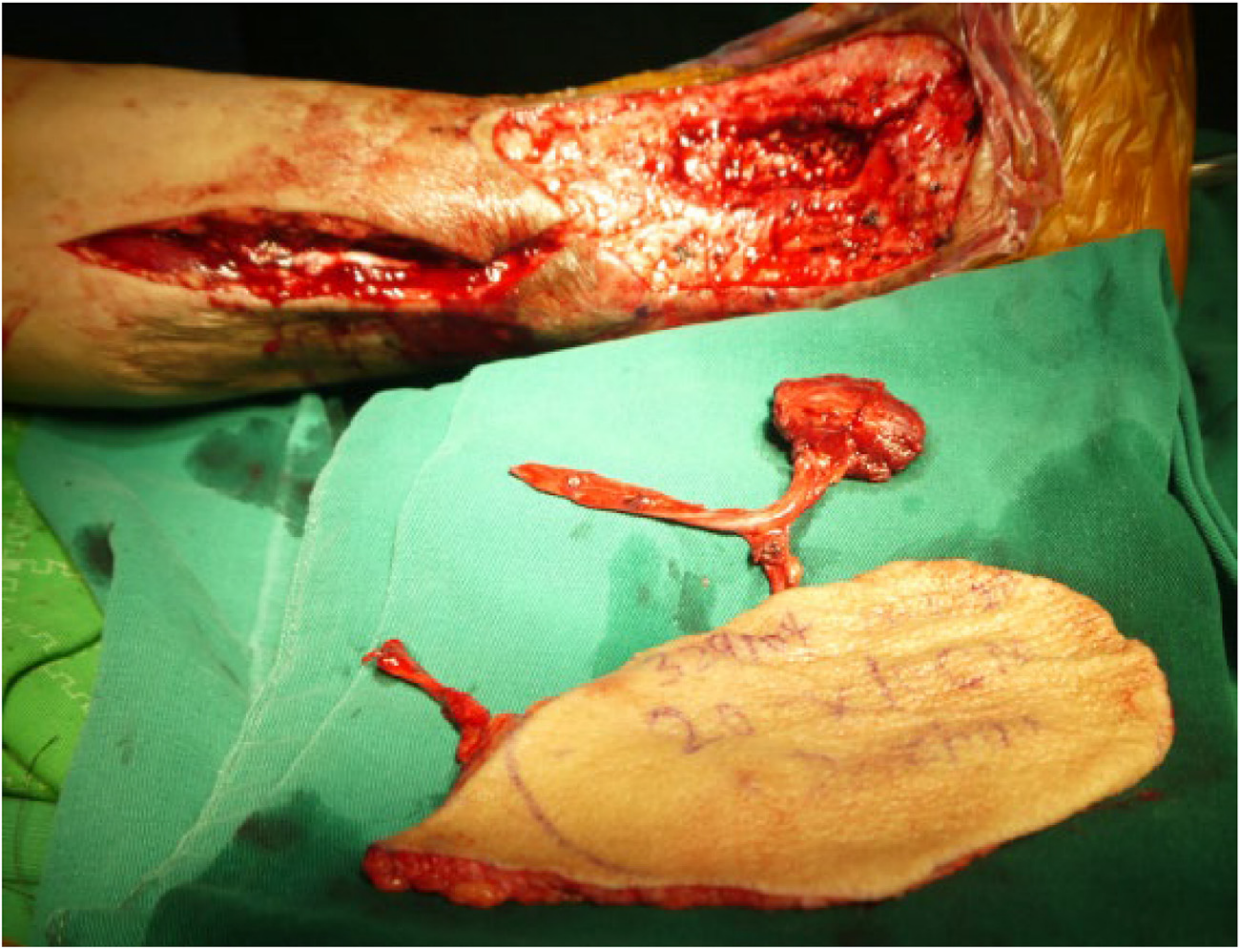
Composite tissue flap transplantation to repair the wound site.

A 58-year-old male patient presented with a closed fracture of the right tibia and fibula because of a traffic accident, subsequently enduring repeated sinus discharge for 3 years after surgery. The patient was admitted to the hospital on August 25, 2013. Before admission, the patient underwent 9 surgical procedures, including internal plate fixation, plate removal and external fixator fixation, lesion removal, and gastrocnemius flap displacement. Upon admission, the patient was diagnosed with anemia and asthenia, and had a 5 cm × 6 cm skin defect at the medial malleolus of the right calf with exposed tibia, thin pus on the wound, and extensive areas of bony scars and skin eczema (Figure 3). Wound secretion analysis indicated the presence of *Pseudomonas aeruginosa* that was sensitive to vancomycin and cephalosporin. X-ray examination revealed decreased bone density in the lower part of the tibia, concomitant with sequestrum formation. Following the administration of sensitive antibiotics to improve the patient's general condition, the osteomyelitis lesion was removed under spinal anesthesia, and the VSD was drained. One week later, the artificial bone materials mixed with vancomycin were implanted into the bone marrow cavity under combined anesthesia (Figure 4). Furthermore, free ALT perforator chimeric composite tissue flap transplantation (flap area 14 cm × 23 cm, muscle flap 4.0 cm × 3.5 cm × 3.5 cm) was performed (Figure 5). The tibia was immobilized using a plaster cast, and anastomosis of the anterior lateral femoral circumflex artery and vein with the posterior tibial artery and vein was carried out, while the other lateral femoral circumflex vein was connected to the great saphenous vein. Following a 10-day drainage period, the tibia was immobilized and protected using a plaster cast for 3 months. Post-surgical wound healing progressed favorably, with callus formation observed on imaging at 2 months post-operatively. Complete tibial healing was achieved after 5 months and the patient began walking with gradually increasing weight on plaster splints after 2 months. No recurrence was detected during the 1-year follow-up period.

## Discussion

Lower limbs trauma is a common medical issue, as evidenced by its prevalence.^14^ Owing to the anatomical features of the lower limbs, including limited soft tissues in the front, middle, and lower parts of the lower limbs along with poor blood supply, these wounds are prone to developing soft tissue defects, bone exposure, and chronic osteomyelitis. Infected bone lesions are surrounded by sclerotic bones with poor blood supply and reactive hyperplasia of the bony shell. Moreover, the surrounding skin, subcutaneous soft tissues, and muscle tissues are scarred. Furthermore, the presence of internal implants complicates the immune system's ability to function effectively, thereby, hindering the control of local infections and impeding the healing process.^15^

The application of free myocutaneous flap transplantation can eliminate the profile and fill the residual bone cavity after the resolution of lesions. Therefore, this technique can be employed to repair soft tissue defects and osteomyelitis simultaneously.^16^ It is a relatively safe and effective method. However, a large volume of tissue is removed with the musculocutaneous flap, damaging the donor site of the flap. The soft tissue in the osteomyelitis area in the lower limbs is often thin before the injury and the volume of the musculocutaneous flap exceeds that required for the repair of the wound, thus resulting in a bloated appearance and limited function after repair.^17^

## Poor condition of the patient

Owing to anemia, malnutrition, diabetes, and other underlying conditions, a comprehensive and accurate assessment of the body and physiological conditions of the patients is required. A comprehensive systemic examination can provide valuable insights into the potential diseases that may compromise the immune system, such as anemia, hypoproteinemia, and diabetes. This information is crucial for clinicians to understand the patient's susceptibility to infections and their ability to tolerate surgical interventions. To identify focal areas of osteomyelitis, imaging techniques such as X-ray, CT, or MRI can be used to determine the extent and severity of bone and soft tissue lesions. The vascular condition of the limbs can be accurately assessed using vascular B-ultrasound, CTA, or DSA. These assessments serve as the foundation for developing a vascular anastomosis plan for free flap transplantation.^18^

## Main points of debridement

The complete debridement procedure is crucial for reducing the bacterial load of the infected foci, and it is also the key to the successful treatment and prevention of recurrent infections.^19^ Therefore, this surgical procedure can be performed to remove the sticking scars and the hardened bone to the greatest extent possible, with a focus on ensuring a healthy blood supply in the osteotomy surface of the cortical bone—a characteristic known as the “red pepper sign” (Paprika sign). Additionally, the wound should be free from inflammatory hyperplastic granulation and inactive tissue. Furthermore, no avascular necrosis skin or crust on the wound margin and no dry necrotic connective tissue should be present. During the treatment process, patients require an average of 2–3 debridements to achieve negative bacterial culture. Overall, the infected foci should be approached as low-grade malignant tumor and radical debridement should be adopted to expand the treatment scope.^20^

## Bacterial culture and drug sensitivity experiment

1. Upon admission to the hospital, it is imperative to conduct thorough wound debridement. According to the Chinese experts’ consensus on the diagnosis and treatment of infection after internal fixation (2018), administering antibiotics prior to debridement is discouraged to optimize the efficacy of intraoperative infection tissue culture and enhance the likelihood of yielding positive results.
2. The “3-2-1″ principle is recommended for the selection of materials and post-operative diagnosis. Specifically, suspected infected tissues should be collected from at least 2 sites during surgery to test for pathogenic bacteria. The diagnosis of infection by the same pathogenic bacteria can be made based on the positive results from the 2 sites. For highly virulent pathogenic bacteria, the culture based on tiny samples can be used to determine the type of bacteria.^18^
3. The bacterial culture and drug sensitivity test of pus and infected tissues should be conducted repeatedly and appropriate antibiotics should be administered in a targeted manner.

### Use of internal implants and external fixation

This group of patients did not use vascularized bone grafting for bone defect repair. During treatment, the patient's internal fixation should be removed as much as possible and replaced with an external fixation frame and plaster. Internal implant removal and retention are contingent upon several factors, including the stability of the internal implant-bone structure, location and duration of infection, physiological state of the host, type and virulence of pathogenic bacteria, and adequacy of soft tissue cleansing.

Patients who underwent treatment for >3 months were assessed for further intervention. If the fracture had fully healed, removal of internal fixation is recommended. Conversely, if the fracture was not healed, retention of internal fixation was warranted. Notably, 1 patient (6%) experienced disease recurrence. In cases where bone instability ensues following debridement, the adoption of a unilateral or circular external fixator may be necessary. When applying an external fixator, it is imperative to position it away from the infected area to facilitate optimal post-operative care.

### Novelty of the proposed method

The soft tissue in the osteomyelitis area of the lower limbs is often thin under physiological conditions before the injury and the volume of musculocutaneous flaps exceeds that required for repairing the wound, resulting in a bloated appearance after repair and limited functions. In 1989, Koshima proposed the concept of perforator flaps with free ALT flaps. Unlike traditional flaps, these flaps do not carry deep fascia; only the perforating vessels supplying blood to the flap are dissected, allowing for various cutting patterns and achieving a medium flap thickness. Among them, the chimeric tissue flap can be used for different perforators supplying skin, muscle, and bone, fulfilling the requirements for comprehensive wound tissue repair.^21^ Accurate tissue repair can be achieved once the repair volume and content are determined.

Moreover, the pathological characteristics of chronic osteomyelitis should be considered during ALT perforator flap transplantation. Preoperative Doppler ultrasound examination of the skin flap donor site, combined with CT angiography or magnetic resonance angiography, aids in identifying larger perforator exit points, facilitating precise flap design during surgery. Additionally, preoperative vascular examination of the recipient area helps in identifying suitable arteries and veins for anastomosis during surgery. Patients with chronic osteomyelitis typically exhibit a prolonged disease course, severe inflammation in the affected limbs, and evident scar hyperplasia. Under the long-term stimulation of chronic inflammation, the blood vessels in the affected area often show pathological changes, such as poor elasticity and uneven intima. Therefore, vascular anastomosis points should be selected from areas that are less affected by inflammation. Prior to vessel division, a clamping test should confirm normal distal limb blood flow, if compromised, the alternative vessels should be selected or flap vessel flow-through technology used to ensure adequate limb blood supply. During ALT perforator flap dissection, a longer vascular pedicle should be preserved whenever possible to position the anastomosis away from the infected wound area. Perforator flaps can extend the length of the vascular pedicle. However, if a chimeric flap (skin flap + muscle flap) needs to be cut out, the relationship between the filling position of the muscle flap and length of the vascular pedicle should be considered. If necessary, vascular transplantation can be used to solve the shortage of the pedicle. Striving for multiple vascular anastomoses is essential, with the flow-through technology serving to facilitate anastomosis and bolster limb blood supply. Moreover, it can aid in identifying the high perforating branches of the lateral femoral circumflex artery, thus augmenting arterial networks. In venous anastomosis procedures, using the large and small saphenous veins should be prioritized owing to their robust walls and consistent anatomical structures. Complementarily, the tibial anterior and posterior tibial arteries can be used alongside these veins. This preference stems from the challenges posed by the thin-walled, inflamed deep veins, which may complicate dissection, separation, and anastomosis. Maintaining an arteriovenous ratio of 1:2 whenever feasible is advisable. In instances where repairing limb blood vessel separation proves challenging, a bridge link of the contralateral limb blood vessel can be used to ensure flap perfusion. In addition, designing skin flap with ample area is essential, as skin defects from osteomyelitis lesions often undergo contracture due to prolonged inflammatory scar hyperplasia. Adequate soft tissue coverage is imperative to accommodate the vascular pedicle appropriately. During the excision of chimeric tissue flaps, attention should be paid to the often diminutive perforating branches supplying the muscle. Cutting a larger flap than necessary during muscle flap excision allows for subsequent trimming to meet tissue repair requirements, while also ensuring a robust blood supply and minimizing dead space.

In this study, all patients had bone defects with diameters <4 cm, which were repaired using biodegradable bone biomaterials. The use of a biodegradable bone biomaterial carrier allowed for a high concentration of antibiotics to be delivered locally. Calcium sulfate, a commonly used bone filler, was particularly effective in increasing local antibiotic concentration.^22^ The advantage of this local treatment approach is that while drug concentration is highly elevated in the treated area, systemic drug exposure and the associated toxic and side effects on important organs are significantly reduced. Additionally, this approach facilitated the filling of bone defects, eliminated dead spaces, and prevented surrounding soft tissues from interfering with new bone growth. Calcium sulfate also has strong osteoinductive osteogenic activity, which is conducive to the repair of bone defects. Owing to its favorable biocompatibility, this material can be degraded and absorbed completely.^23^ Vancomycin was found to exert >73% of its activity in calcium sulfate. High-level release responses were observed from the first day after implantation, providing a stable local antibiotic concentration for up to 4 weeks. Based on drug sensitivity testing, this technique could be an excellent option for administering topical antibiotics.^24^

### Advantages and limitations of the ALT flap compared to other reconstructive techniques

When selecting the ALT flap during surgery, its vascular pedicle is long and can be anastomosed away from the infected area, making it safer. The skin flap has a large cutting area and can be combined with the muscle flap to form a chimeric skin flap, which has a good effect on filling the dead space. The nutrient vessels of the skin flap can be cut and anastomosed with the flow of the donor blood vessels, without affecting the distal blood supply of the donor area. The vascular network of the donor site can be reconstructed in case of segmental damage to the original blood vessels. The SCIP flap is thinner and more aesthetically pleasing than the ALT flap, with a more concealed supply area. However, its vascular pedicle is short and thin, making it difficult to operate and carry muscles to form a composite tissue flap.

In conclusion, chronic osteomyelitis of the lower limbs with soft tissue defects is a major problem that has plagued orthopedic surgeons for a long time. The ALT perforator flap transplantation has been identified as a feasible treatment method. This treatment can improve the condition of patients with osteomyelitis. The muscle flap fills the dead space and medullary cavity and skin flap covers the skin defect. For patients with bone defects, the calcium sulfate artificial bone in combination with vancomycin may prove to be an effective therapeutic option. Nevertheless, further clinical research is needed to compare its efficacy to that of the Masquelet technology.

## Declaration of competing interest

The authors declare that there are no conflict of interest.
